# Heterologous Expression of Thanatin Using Dual Transposon System in *Saccharomyces cerevisiae*

**DOI:** 10.3390/biology15151310

**Published:** 2026-08-05

**Authors:** Song Li, Jia Song, Bo Sun, Kuanbo Liu, Ruimin Li, Bin Xiang, Hui Zhang, Chen Zhao

**Affiliations:** 1 College of Chemical Engineering, Shenyang University of Chemical Technology, Shenyang 110142, China; 13795110298@163.com; 2 Key Laboratory of Grain and Oil Biotechnology, Academy of National Food and Strategic Reserves Administration, Beijing 100037, China; songj@ags.ac.cn (J.S.); sunb@ags.ac.cn (B.S.); lkb@ags.ac.cn (K.L.); lrm@ags.ac.cn (R.L.); 3 CommBio Therapeutics Co., Ltd., Shanghai 201203, China; bin.xiang@commbiotx.com; 4 College of Environmental and Safety Engineering, Shenyang University of Chemical Technology, Shenyang 110142, China

**Keywords:** thanatin, PiggyBac transposon, Ty2 transposon, *Saccharomyces cerevisiae*, heterologous expression, antibacterial activity

## Abstract

Thanatin is a small cationic antimicrobial peptide with broad-spectrum antibacterial activity, considered a promising candidate to combat the global antibiotic resistance crisis. Microbial biosynthesis represents an attractive strategy for sustainable production of antimicrobial peptides. In this study, we constructed a heterologous expression system for thanatin in *Saccharomyces cerevisiae* CENPK2 using both PiggyBac (PB) and Ty2 retrotransposon systems, demonstrating the utility of yeast as a host for peptide production and transposon-based genetic engineering. By designing transposon-based expression plasmids incorporating the CL1 selection tag, we achieved efficient expression of thanatin. The expression of thanatin-derived peptides was confirmed by Tris-tricine-SDS-PAGE and liquid chromatography-mass spectrometry (LC-MS). In vitro antibacterial assays demonstrated that the fermentation supernatant of engineered yeast significantly inhibited the growth of *Escherichia coli* O157: H7, *Salmonella* typhimurium, *Aeromonas veronii* (JL-2)*,* and *Acinetobacter baumannii*. Notably, strains harboring both PB and Ty2 systems exhibited stronger antibacterial activity than those with a single system. Scanning electron microscopy further revealed that thanatin caused damage to bacterial cell membranes and led to cell lysis. Hemolysis tests proved that the physiological saline-replaced fermentation supernatant possessed low hemolytic activity. This study successfully established a transposon-based platform for the heterologous expression of thanatin.

## Simple Summary

Bacteria that no longer respond to existing antibiotics pose a serious threat to human health. There is an urgent need for new weapons against these “superbugs.” One promising weapon is a natural small protein called thanatin, which can kill many harmful bacteria. However, producing thanatin in large amounts is difficult and costly. In this study, we turned ordinary baker’s yeast into a tiny factory to produce thanatin. We used two genetic tools (like molecular scissors and copiers) to insert the thanatin gene permanently into the DNA of the yeast. The engineered yeast successfully produced and secreted thanatin into the surrounding culture medium. When we tested this liquid against four dangerous bacteria, including those causing food poisoning and hospital-acquired infections, it strongly stopped their growth. Importantly, the produced thanatin did not harm red blood cells, suggesting it is safe for potential medical use. Our work shows that a common food-grade microorganism can be turned into a production system for a powerful antibacterial agent. This offers a natural, safe, and potentially low-cost strategy to fight drug-resistant infections and protect public health.

## 1. Introduction

Antimicrobial peptides (AMPs) are small, cationic, amphipathic molecules that serve as essential components of the innate immune system. They exhibit broad-spectrum activity against Gram-negative and Gram-positive bacteria, fungi, parasites, and both enveloped and non-enveloped viruses through multiple mechanisms, including disruption of the viral envelope, interaction with capsid proteins, blocking of cellular entry, inhibition of viral replication, and modulation of the host immune response, and are considered promising candidates to combat the global antibiotic resistance crisis [1,2]. Thanatin, a 21-amino-acid antimicrobial peptide (GSKKPVPIIYCNRRTGKCQRM), adopts a β-hairpin structure stabilized by a single disulfide bond between Cys11 and Cys18. It was first isolated from the hemolymph of the hemipteran insect *Podisus maculiventris* (the spined soldier bug) following immune challenge [3]. Thanatin offers several advantages for therapeutic development: it displays potent activity against a wide range of Gram-negative and Gram-positive bacteria [4]. In the lipopolysaccharide (LPS) transport system of Gram-negative bacteria, LptA is a critical periplasmic protein that bridges the inner-membrane ABC transporter LptB_2_FGC and the outer-membrane translocon LptD/E. By shielding the hydrophobic lipid A moiety during transit, LptA ensures the safe passage of LPS across the aqueous periplasmic space. Thanatin exerts its antibacterial action by targeting both LptA and LptD within this essential machinery, thereby interfering with LPS transport and disrupting outer membrane biogenesis [5]. Furthermore, its low hemolytic activity supports its potential as an alternative to conventional antibiotics [6].

Because biological extraction yields only small amounts of thanatin and involves complex procedures, production relies primarily on chemical synthesis or heterologous expression. However, chemical synthesis remains expensive, difficult to scale up, and environmentally unfriendly [7]. The *Saccharomyces cerevisiae* expression system offers the advantages of a eukaryotic host, including safety, lack of pollution, and the potential for high-level expression under strong promoters.

**Figure 1:**
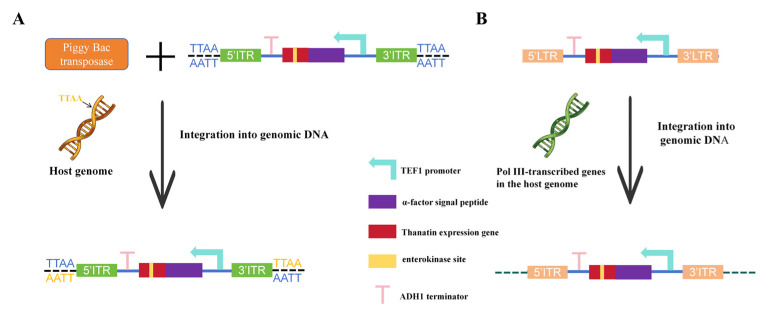
Schematic diagram of the PiggyBac transposon system and Ty retrotransposon system. (**A**) Schematic diagram of the PiggyBac transposon system. (**B**) Schematic diagram of the Ty retrotransposon system.

*S. cerevisiae* harbors an endogenous Ty retrotransposon system capable of efficient transposition [8]. Ty elements are structurally similar to retroviruses but are non-infectious and transpose via a copy-and-paste mechanism [9]. The PiggyBac (PB) transposon, originally discovered in the cabbage looper moth *Trichoplusiani* [10], operates through a cut-and-paste mechanism [11]. With the PB transposase (PBase), the transposon is precisely excised from the donor site through recognition of the inverted terminal repeats (ITRs) and re-integrates into a TTAA target site without leaving a footprint [12]. The PB transposon exhibits high transposition activity and favorable genome-wide integration site distribution [13]. The schematic diagrams of the PiggyBac (PB) and Ty2 transposon systems are shown in Figure 1.

CL1 is a degradation signal in *S. cerevisiae* that directs fused proteins for ubiquitination via the Ubc6p/Ubc7p pathway and subsequent proteasomal degradation [14]. LEU2 and KlURA3 are commonly used selectable markers in *S. cerevisiae*. Fusing them with CL1 enables counterselection against low-copy integrants and facilitates the screening of strains carrying multiple copies of the target gene through simple growth selection [15]. The Ty2 transposon system has been successfully employed for heterologous gene expression in *S. cerevisiae* [16], and the PB transposon system has enabled stable recombinant protein production in the probiotic yeast *Saccharomyces boulardii* [17]. However, the combined application of these two transposon systems for antimicrobial peptide production has not been reported.

In this study, we introduced the thanatin-bearing PB transposon system and the Ty2 system into the host strain CENPK2, employing a LEU2-CL1 fusion (for PB) and a KlURA3-CL1 fusion (for Ty2) as selection markers, to achieve heterologous expression of thanatin in *S. cerevisiae*. We subsequently evaluated the in vitro antimicrobial activity of the expressed product against various pathogenic bacteria and its hemolytic activity toward mammalian erythrocytes.

## 2. Materials and Methods

### 2.1. Strains, Plasmids, and Media

*Escherichia coli* Top10 for plasmid construction, *S. cerevisiae* CENPK2 as the experimental host strain, *E. coli* O157:H7 (ATCC K88), *Salmonella* Typhimurium (ATCC 9027), *Aeromonas veronii* JL-2 (ATCC 13124), and *Acinetobacter baumannii* (ATCC 19606) for antibacterial activity assays.

pUC57, pESCgp04-HIS, and pBluescript as cloning vectors and pESC-LEU providing the LEU2 selection marker.

Yeast cells were cultured in YPD medium containing 10 g/L yeast extract, 20 g/L peptone, and 20 g/L glucose. For solid medium, 20 g/L agar was added.

*E. coli* O157:H7, *S.* Typhimurium, and *A. veronii* JL-2 were cultured in LB medium containing 5 g/L yeast extract, 10 g/L peptone, and 10 g/L NaCl. For solid medium, 20 g/L agar was added.

*A. baumannii* was cultured in TSB medium containing 17.0 g/L casein peptone (pancreatic digest), 3.0 g/L soy peptone, 5.0 g/L NaCl, 2.5 g/L glucose, and 2.5 g/L dipotassium phosphate. For solid medium, 20 g/L agar was added.

Restriction endonucleases were purchased from New England Biolabs (NEB, Ipswich, MA, USA). Plasmid extraction kit was purchased from Genstar (Beijing, China). All other chemical reagents were of analytical grade. 

**All primers used in this study are listed in** **Table S1** **.**

### 2.2. Synthetic Thanatin

Synthetic thanatin (purity > 95%) was purchased from Synpeptide Co., Ltd. (Nanjing, China).

### 2.3. Expression Vector Design and Construction

**Figure 2:**
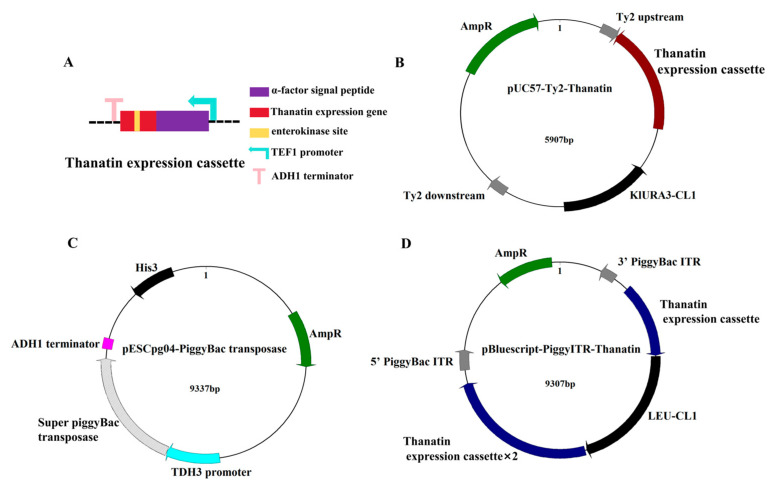
Schematic diagrams of all plasmid constructions designed in this experiment and the thanatin expression cassette. (**A**). Schematic diagram of the thanatin expression cassette. (**B**). Map of plasmid pUC57-Ty2-KlURA3-CL1. (**C**). Map of plasmid pESCgp04-HIS-PiggyBac transposase. (**D**). Map of plasmid pBluescript-PiggyBac-thanatin.

Several key modules were prepared for vector construction, including the promoters TEF1 and TDH3, the α-factor signal peptide, and the ADH1 terminator. The selection markers URA3 and LEU2, each fused to the CL1 degradation signal, were employed. The target gene consisted of two tandem thanatin sequences linked by an enterokinase cleavage site. All genetic elements were amplified with primers containing homologous recombination sequences, and the PCR products were gel-purified and used for Gibson assembly. The schematic diagram of the thanatin heterologous expression cassette is shown in Figure 2A.

#### 2.3.1. Construction of the Vector Based on Ty2 Retrotransposons

Ty retrotransposon sequences have been widely used as integration loci for heterologous gene expression in yeast. In this study, primers Ty2-ITR-F and Ty2-ITR-R were designed based on the *S. cerevisiae* Ty2 consensus sequence defined by Jérôme Maury et al. [18]. Genomic DNA of *S. cerevisiae* CENPK2 was used as the template to amplify the Ty2 long terminal repeat (LTR) sequence. The obtained LTR fragment was then amplified with two primer pairs (Ty2-upstream-F(Gib)/Ty2-upstream-R(Gib) and Ty2-downstream-F(Gib)/Ty2-downstream-F(Gib)) to generate the upstream and downstream homologous arms, respectively.

Subsequently, overlapping PCR was performed to insert KlURA3-CL1 between the upstream and downstream homologous arms, and the fused fragment was assembled into the linearized pUC57 backbone by Gibson assembly, yielding the universal integration plasmid pUC57-Ty2- KlURA3-CL1.

An expression cassette comprising the promoter, α-factor signal peptide, tandem thanatin genes, and terminator was then constructed by sequential assembly. This cassette was amplified with primers pTEF1-F(Gib) and tADH1-R(Gib) and inserted between the 5′ upstream homologous arm and the *KlURA3* gene of the linearized pUC57-Ty2-KlURA3-CL1 plasmid by Gibson assembly, generating the final recombinant expression plasmid pUC57-Ty2-thanatin (Figure 2B).

#### 2.3.2. Construction of the Vector Based on PiggyBac Transposons

##### Construction of pESCpg04-PiggyBac Transposase Plasmid

To construct the PiggyBac transposase expression vector, the coding sequence for the hyperactive PiggyBac transposase was amplified by PCR using primers Tpos-F(Gib) and Tpos-R(Gib) from a plasmid containing the sequence described by Corey Brizzee et al. [18]. The resulting PCR product was inserted into the pESCgp04-HIS vector by Gibson assembly, and the assembled plasmid was transformed into competent cells, yielding the recombinant plasmid pESCgp04-HIS-PiggyBac transposase (Figure 2C).

##### Construction of pBluescript-PiggyITR-Thanatin

For the PiggyBac inverted terminal repeat (ITR) constructs, the 3′ ITR plus insulator and 5′ ITR plus insulator fragments were amplified by PCR using primers PB-3′ ITR-F(Gib)/PB-3′-ITR-R(Gib) and PB-5′ITR-F(Gib)/PB-5′-ITR-R(Gib), respectively, with synthetic gene fragments as templates. The ITR sequences are essential for transposase recognition and transposition, and the insulator fragment enhances transposition stability and expression efficiency. To construct the final expression plasmid, the LEU2-CL1 fragment was first inserted between the upstream and downstream homologous arms of the PiggyBac transposon via overlapping PCR. The fused fragment was then assembled into the linearized pBluescript backbone by Gibson assembly, yielding the universal integration plasmid pBluescript-PiggyBac-LEU2-CL1.

Expression cassettes were constructed by sequentially assembling the promoter, α-factor signal peptide, tandem thanatin genes, and terminator, generating three cassettes (Than1, Than2, and Than3). Than1 was inserted between the 5′ITR homologous arm and LEU2-CL1, and Than2 and Than3 were inserted between LEU2-CL1 and the 3′ITR homologous arm of the linearized pBluescript-PiggyBac plasmid by Gibson assembly, resulting in the recombinant expression plasmid pBluescript-PiggyBac-thanatin (Figure 2D).

### 2.4. Yeast Transformations and Thanatin Production

*S. cerevisiae* CENPK2 competent cells were prepared using the lithium acetate method [19]. To construct the Ty2 system strain, 1 µg of linearized pUC57-Ty2-thanatin was transformed into CENPK2 competent cells. To construct the PB system strain, 1 µg of linearized pBluescript-PiggyBac-thanatin and 1 µg of pESCgp04-HIS-PiggyBac transposase were co-transformed into CENPK2 competent cells. For the dual-system (Ty2 + PB) strain, the same aforementioned plasmids were co-transformed into competent cells prepared from a pre-existing Ty2 transformant. In each transformation, the DNA mixture was added to 100 µL of competent cells along with 5 µL Salmon sperm DNA and 500 µL PEG/LiAc solution. After heat shock at 42 °C for 15 min [20], cells were recovered and plated on synthetic complete medium lacking histidine and leucine (SC-His-Leu) or lacking uracil, histidine, and leucine (SC-Ura-His-Leu) as appropriate. Positive transformants were selected after 2–4 days of incubation at 29 °C.

For fermentation, single colonies of the confirmed transformants were inoculated into YPD broth and grown overnight at 29 °C with shaking at 220 rpm. The overnight cultures were then inoculated into 100 mL of fresh YPD medium at an initial OD_600_ of 0.1 and cultivated at 29 °C, 220 rpm, for 5 days. OD_600_ was measured every 24 h to monitor cell growth. All fermentations were performed in three independent biological replicates (*n* = 3). Data are presented as mean ± standard deviation (SD). Statistical comparisons of cell growth among the different strains were performed using one-way analysis of variance (ANOVA) followed by Tukey’s post hoc test, with a *p*-value < 0.05 considered statistically significant. Culture supernatants were harvested by centrifugation at 12,000× *g* for 10 min at 4 °C and stored at −20 °C for subsequent analyses.

### 2.5. Shake-Flask Fermentation of Transformants

Positive colonies were streaked on YPD plates for activation. Single colonies were then inoculated into YPD broth and grown overnight. For transformants carrying the Ty2 system, the PB system or both systems, 1% (*v*/*v*) of the overnight seed culture was inoculated into 100 mL YPD medium and incubated at 29 °C with shaking at 220 rpm for 5 days. OD_600_ was measured every 24 h to assess the growth effects of the different transposon systems.

### 2.6. Tris-Tricine-SDS-PAGE Analysis

The fermentation broth was centrifuged (4 °C, 12,000 rpm, 10 min), and the supernatant was re-centrifuged under the same conditions. A 500 μL aliquot of the supernatant was concentrated to 100 μL using a 3 kDa cutoff centrifugal filter (4500 g, 25 min). Discontinuous gels (4% stacking, 15.5% spacer, and 15.5% separating) were prepared. Samples (40 μL sample supplemented with 10 μL 5× loading buffer) were boiled, centrifuged, cooled, and then loaded (12 μL per lane) alongside 5 μL of protein marker. Electrophoresis was performed at 30 V for 1 h through the stacking gel and at 100 V for 4 h through the spacer and separating gels. Gels were stained with ExBlue stain and imaged.

### 2.7. Experimental Method for Quantitative Determination of Thanatin in Fermentation Supernatant by High-Performance Liquid Chromatography (HPLC)

For quantitative determination of antimicrobial peptide concentration via HPLC, the tandem peptide present in the fermentation broth was initially digested with recombinant enterokinase (P4237-1000U), followed by quantification against a standard curve generated from chemically synthesized antimicrobial peptide standards at serial concentrations.

HPLC analysis was performed on a Thermo Fisher UltiMate 3000 HPLC (Thermo Fisher Scientific, Waltham, MA, USA) system equipped with a UV detector. Chromatographic separation was achieved on a C18 column (250 mm × 4.6 mm, 5 μm particle size). The detection wavelength was set to 220 nm, and the column temperature was maintained at 30 °C. The solvent system consisted of loading solvent (2% acetonitrile in water containing 0.1% formic acid), mobile phase A (water containing 0.1% formic acid), and mobile phase B (acetonitrile containing 0.1% formic acid). Samples were loaded onto a trap column at 30 °C using a temperature-controlled autosampler, with loading solvent as the mobile phase at a flow rate of 2 µL/min. After a 5 min loading period, the sample was eluted onto the analytical column. The flow rate on the analytical column was set to 0.3 µL/min, and the following gradient elution program was applied: 0–22 min, solvent B increased from 5% to 38%; 22–26 min, solvent B increased from 38% to 80%; 26–32 min, solvent B decreased from 80% to 5%, using Chromeleon nonlinear gradient curve 5. Chromatographic separation was carried out at 30 °C. The concentration of the target peptide in the fermentation supernatant was calculated using a standard curve generated from serial dilutions of the synthetic thanatin standard (purity > 95%). All HPLC analyses were performed in three independent biological replicates, and each replicate was measured in duplicate. The values are expressed as mean ± standard deviation (SD). Note that the quantified product is the EK-tag-free fragment released from the tandem construct after enterokinase cleavage.

### 2.8. LC-MS/MS Identification

#### 2.8.1. Enzyme Digestion and Mass Spectrometry Identification

The protein bands were destained, washed, reduced with 10 mM DTT at 37 °C for 30 min, and alkylated with 50 mM IAA in the dark for 15 min. After dehydration, the gel pieces were digested overnight at 37 °C with proteomics-grade trypsin (0.01 µg/µL; purchased from Promega, Madison, WI, USA) in 50 mM NH_4_HCO_3_ containing 10% ACN. The resulting peptides were extracted with 67% ACN containing 2% formic acid, concentrated by vacuum centrifugation, and stored at −20 °C until analysis.

#### 2.8.2. Liquid Chromatography–Tandem Mass Spectrometry (LC-MS/MS) Analysis

The digested peptide samples were reconstituted in mobile phase A (0.1% formic acid in water) and analyzed on an UltiMate 3000 RSLCnano system (Thermo Fisher Scientific, Waltham, MA, USA) coupled to a Q Exactive Plus mass spectrometer equipped with a Nano Flex ion source (Thermo Fisher Scientific). Peptides were loaded onto a nanoViper C18 trap column (3 µm, 100 Å; 75 µm × 2 cm) and desalted with 20 µL of mobile phase A. Separation was performed on an Acclaim PepMap RSLC C18 analytical column (2 µm, 100 Å; 75 µm × 25 cm; Thermo Fisher Scientific) at a flow rate of 300 nL/min, with a linear gradient from 5% to 40% mobile phase B (80% ACN, 0.1% formic acid) over 15 min. The column temperature was maintained at 40 °C.

Mass spectrometric detection was carried out on a Q Exactive Plus instrument in data-dependent acquisition (DDA) mode. The spray voltage was set to 1.9 kV, and the ion transfer tube temperature was 320 °C. Full MS scans were acquired at a resolution of 70,000 over a mass range of 350–1500 *m*/*z*, with a maximum injection time of 100 ms. For each DDA cycle, up to 20 MS/MS spectra were collected for precursor ions with charge states of 2+ to 5+. MS/MS scans were acquired at a resolution of 17,500, with a maximum injection time of 50 ms and a normalized collision energy of 28 eV for higher-energy collisional dissociation (HCD). Dynamic exclusion was set to 25 s.

#### 2.8.3. Database Search and Analysis

The raw MS data were processed using Proteome Discoverer 2.5 software (Thermo Fisher Scientific). Searches were performed against a protein database using trypsin as the enzyme, allowing up to two missed cleavages. The precursor mass tolerance was set to 10 ppm, and the fragment mass tolerance was set to 0.05 Da. Carbamidomethylation of cysteine was set as a fixed modification; N-terminal acetylation and oxidation of methionine were set as variable modifications.

### 2.9. Antibacterial Activity Assay

Indicator strains (*E. coli* O157:H7 (ATCC K88), *S.* Typhimurium (ATCC 9027), *A. veronii* JL-2 (ATCC 9027), *A. baumannii* (ATCC 19606)) were cultured overnight in LB or TSB (for *A. baumannii*) at 37 °C and 220 rpm. Overnight cultures were diluted in fresh medium to an OD_600_ of 0.01, and 100 μL aliquots were dispensed into a 96-well plate. Negative control wells contained medium only, and positive control wells contained bacteria without treatment. The fermentation supernatant was concentrated 10-fold using a 3 kDa filter, and 100 μL of the concentrate was added to the experimental wells. The plate was incubated at 37 °C for 12 h, and OD_600_ was measured. To rule out any background antimicrobial activity from the yeast host or the vector backbone, a control strain carrying the empty vector (vector backbone without the thanatin expression cassette) was tested in preliminary experiments in our laboratory. No inhibitory activity has been detected, and the growth profile of this control strain has been found indistinguishable from that of the wild-type CENPK2 strain under the same conditions. On the basis of the previous results, the empty-vector control was omitted from the subsequent main experiments, and the parental strain supernatant was used as the negative control instead.

**The inhibition rate was calculated as Inhibition Rate (%) = [(OD_Control − OD_Test)/(OD_Control − OD_Blank)] × 100.**

The synthetic thanatin standard was dissolved in sterile deionized water to prepare a stock solution at a concentration of 1024 µg/mL, which was then sterilized by filtration through a 0.22 µm filter. The fermentation supernatant was concentrated ten-fold and then diluted to 128 µg/mL, followed by sterilization through a 0.22 µm membrane. The concentration of the fermentation supernatant was determined by HPLC. The values are expressed as the effective peptide concentration (µg/mL) in the concentrated supernatant samples. The indicator strains (*E. coli* O157:H7, *S. typhimurium*, *A. veronii* JL-2, *A. baumannii*) were incubated overnight at 37 °C (16–18 h). The overnight cultures were then transferred to fresh medium and incubated at 37 °C with shaking at 200 rpm until the mid-logarithmic growth phase (OD_600_ ≈ 0.4–0.6). The bacterial suspension was diluted 100-fold to obtain a final inoculum of approximately 1–2 × 10^6^ CFU/mL. For the two-fold serial dilution procedure. For the two-fold serial dilution procedure, 100 µL of the highest-concentration sample solution (prepared at 2× the final desired concentration) was added to the first well of each row in a 96-well microtiter plate. Wells 2 through 11 were each filled with 100 µL of sterile culture medium, while well 12 was left empty. A 100 µL aliquot was then transferred from the first well to the second well and mixed thoroughly; subsequently, 100 µL was serially transferred from the second well to the third, and so on down to the 11th well, resulting in a two-fold dilution series across the row. After mixing, 100 µL was discarded from the 11th well to maintain a consistent volume of 100 µL in each well. Next, 100 µL of bacterial suspension was added to each well from wells 1 through 11, yielding a final volume of 200 µL per well. Positive control wells contained 100 µL of bacterial suspension plus 100 µL of ampicillin solution at a concentration of 256 µg/mL. Blank control wells contained 100 µL of sterile water plus 100 µL of culture medium. All experiments were performed in triplicate. The 96-well plate was incubated at 37 °C for 16–20 h, after which the OD_600_ was measured using a microplate reader. The minimum inhibitory concentration (MIC) was defined as the lowest peptide concentration at which no increase in absorbance (OD_600_) was observed. For MBC determination, aliquots were taken from the MIC well, one to two wells above the MIC, and one to two wells below the MIC. From each selected well, 10–50 µL of the culture was spread onto agar plates, which were then incubated at 37 °C for 18–24 h. The minimum bactericidal concentration (MBC) was defined as the lowest sample concentration that resulted in no visible colony growth on the agar plates. As a negative control, the fermentation supernatant from the parental CENPK2 strain (without integration of the peptide expression cassette) was processed in parallel and tested under the same conditions.

### 2.10. Observation of Bacterial Cell Morphology After Treatment with Fermentation Supernatant by Scanning Electron Microscopy (SEM)

For SEM observation, indicator bacteria were first cultured to mid-logarithmic phase (OD_600_ ≈ 0.4). Cells from 600 μL of each bacterial culture were harvested by centrifugation (4000 rpm, 10 min) and gently washed twice with sterile PBS. The cell pellets were then resuspended in 600 μL of 10-fold concentrated fermentation supernatant and incubated at 37 °C with shaking at 200 rpm for 3 h.

After incubation, the bacterial cells were pelleted by centrifugation, washed twice with PBS, and fixed overnight at 4 °C with 2.5% (*v*/*v*) glutaraldehyde in PBS. Fixed cells were then washed twice with PBS and dehydrated through a graded ethanol series (30%, 50%, 70%, 85%, 95%, and twice with 100% ethanol, 10 min each step). The dehydrated samples were air-dried, freeze-dried, sputter-coated with gold, and observed under a scanning electron microscope (SEM). The volume and OD_600_ of bacterial cultures were standardized across all experimental groups to ensure comparability. All experiments were performed in at least three independent biological replicates, and representative images from each group are shown.

### 2.11. Hemolysis Assay

Rabbit erythrocytes were used for the assay. The 10-fold concentrated fermentation supernatants from PB, Ty2 and PB + Ty2 recombinant yeast strains were buffer-exchanged with physiological saline and used as the test groups. Triton X-100 (1%) served as the positive control (100% hemolysis), and physiological saline served as the negative control (0% hemolysis). Samples were mixed with erythrocyte suspension and incubated at 37 ± 0.5 °C for 4 h. After centrifugation, 100 μL of supernatant was transferred to a 96-well plate, followed by OD_540_ measurement.

**Hemolysis rate was calculated as Hemolysis Rate (%) = [(OD_540_** **Test − OD_540_** **Negative)/(OD_540_** **Positive − OD_540_** **Negative)] × 100.**

## 3. Results

### 3.1. Expression of the Recombinant Thanatin in S.cerevisiae

**Figure 3:**
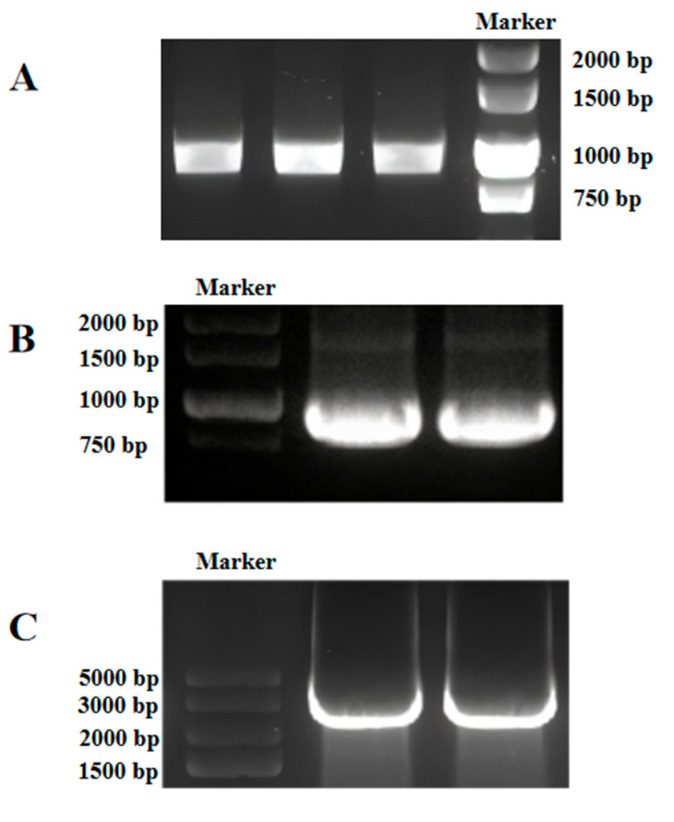
PCR confirmation of the recombinant strains by agarose gel electrophoresis. (**A**). PCR verification of pUC57-Ty2-thanatin. (**B**). PCR verification of PiggyBac transposons. (**C**). PCR verification of pBluescript-piggyITR-thanatin.

The plasmid pUC57-Ty2-thanatin was transformed into CENPK2, and transformants were selected on SC-Ura plates. PCR-verified positive transformants were designated as the Ty2-integrated strain (Ty2). pESCgp04-HIS-PiggyBac transposase and pBluescript-PiggyBac-thanatin were co-transformed into CENPK2 and the Ty2 strain, respectively, selected on SC-His-Leu and SC-Ura-His-Leu plates. Positive transformants were verified by PCR and designated as the PB-integrated strain (PB) and the dual-integration strain (PB + Ty2). These results demonstrate that both the PB and the combined Ty2/PB transposon systems can be efficiently introduced into *S. cerevisiae*, providing a reliable platform for heterologous expression of thanatin. The PCR confirmation results for the recombinant strains are presented in Figure 3.

#### 3.1.1. Tris-Tricine-SDS-PAGE and LC-MS/MS Identification Results

**Figure 4:**
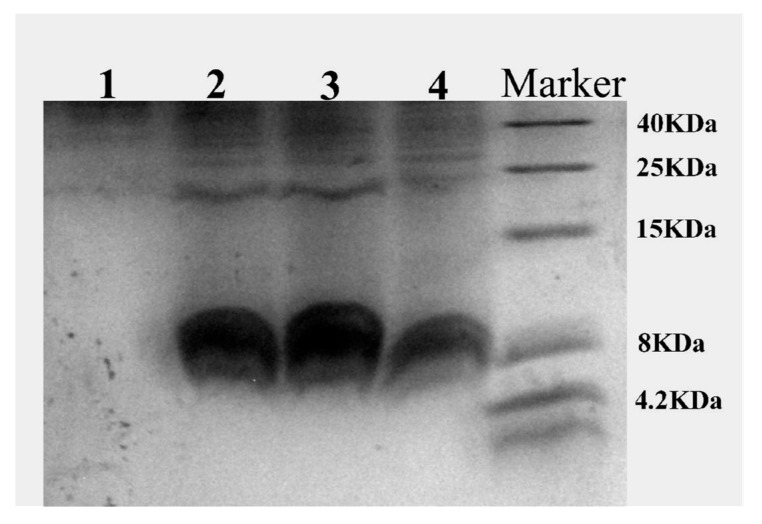
Tris-tricine-SDS-PAGE analysis of fermentation supernatant. 1-CENPK2; 2-Ty2; 3-PB; 4-PB + Ty2.

As shown in Figure 4, an additional protein band between 4 and 8 kDa was detected in the concentrated fermentation supernatants from the PB, Ty2 and PB + Ty2 strains compared with the CENPK2 control. The theoretical molecular weight of monomeric thanatin is approximately 2.4 kDa. In this study, a 2 × thanatin construct (two copies linked by an enterokinase cleavage site) was expressed, with an expected molecular weight of approximately 5.4 kDa. The observed 8 kDa band on the gel may reflect post-translational modifications or aggregation of the expressed peptide. The observed band is consistent with the expected value, indicating successful secretion of the target peptide into the culture medium, which facilitates downstream purification and application.

Notably, the expression cassette contained a LEU2-CL1 fusion selection marker, a design that theoretically enables the enrichment of multicopy integrators through counterselection. In practice, all engineered strains exhibited robust growth (Figure 7) and successfully secreted thanatin into the supernatant. These results suggest that the CL1-based selection strategy is effective in supporting high-yield fermentation without compromising host fitness. In terms of application, this approach could be directly incorporated into strain engineering pipelines for industrial production, where both high copy number and host vitality are critical.

**Figure 5:**
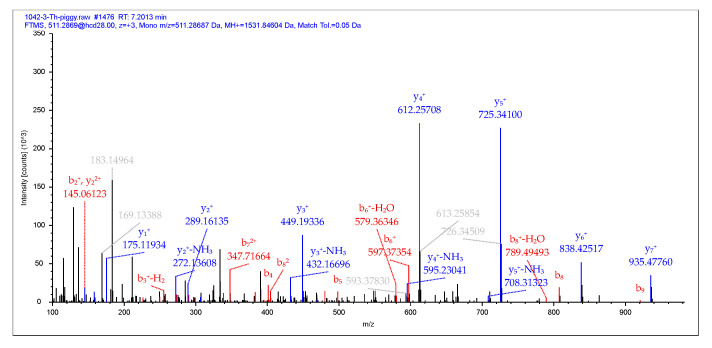
MS/MS spectrum of the target protein from the Ty2 experimental group.

**Figure 6:**
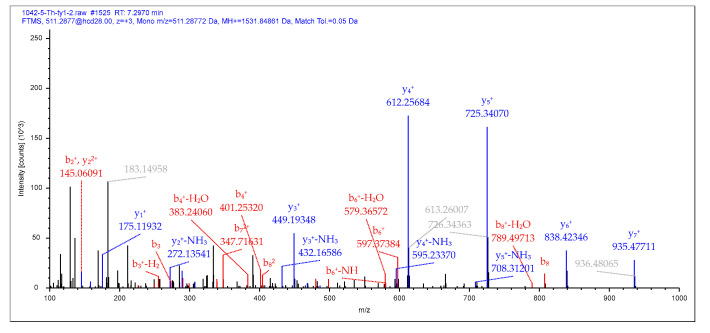
MS/MS spectrum of the target protein from the PB experimental group.

**Figure 7:**
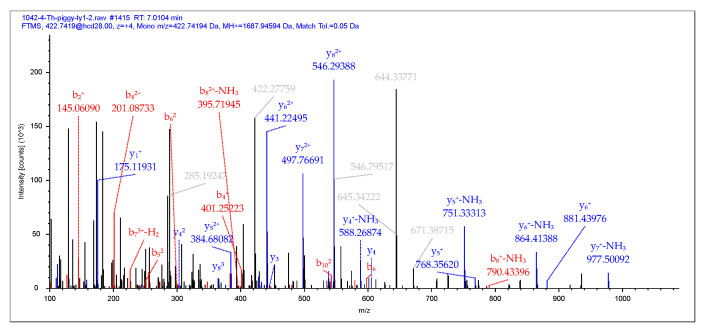
MS/MS spectrum of the target protein from the Ty2 + PB experimental group.

As shown in Figure 5, Figure 6 and Figure 7 LC-MS/MS analysis of the target bands from the Ty2, PB, and PB + Ty2 strains identified thanatin-specific peptide sequences (Ty2 and PB: GSKKPVPIIYCNR; PB + Ty2: GSKKPVPIIYCNRRT). These data confirm that thanatin-derived peptides were successfully expressed in all three engineered strains. The detection of these specific sequences by high-resolution mass spectrometry provides evidence for the expression of the thanatin gene cassette and supports the reliability of the transposon-based expression strategy for peptide production.

#### 3.1.2. Effect of Different Transposon Systems and Thanatin Expression on Yeast Growth

**Figure 8:**
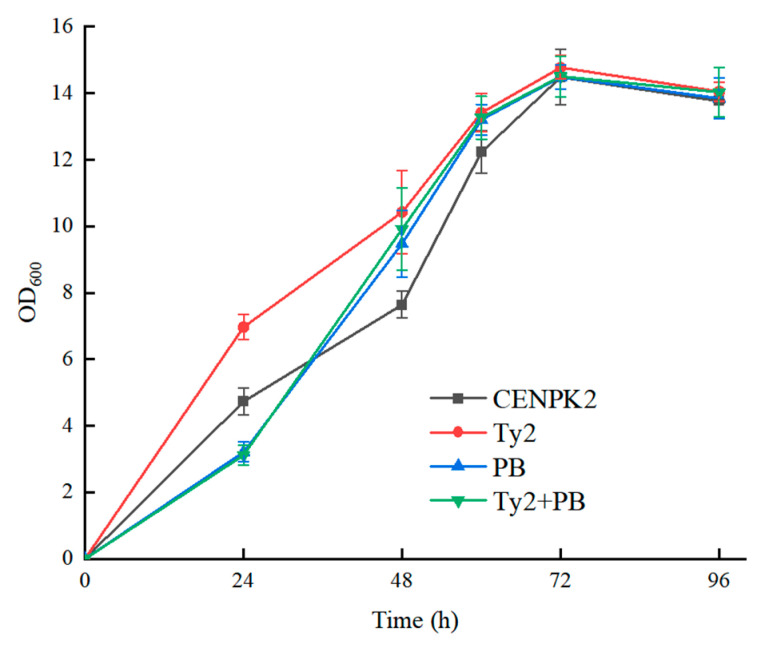
Growth curves of different strains.

Growth curves of the CENPK2, Ty2, PB and PB + Ty2 strains in YPD medium are shown in Figure 8. All strains displayed similar growth profiles and reached maximum biomass at approximately 72 h. The maximum OD_600_ values were 14.48 (CENPK2), 14.77 (Ty2), 14.49 (PB), and 14.5 (PB + Ty2), indicating that neither the transposon systems nor thanatin expression significantly affected host growth under the tested conditions. These data demonstrate that the metabolic burden imposed by the genetic modifications is minimal, confirming that the engineered strains maintain growth performance largely comparable to that of the wild-type host. From an application perspective, this growth robustness is an important characteristic for fermentation-based production, as it supports the use of simple culture media and standard cultivation protocols, and facilitates process scale-up.

### 3.2. Quantitative HPLC Analysis Results

**Figure 9:**
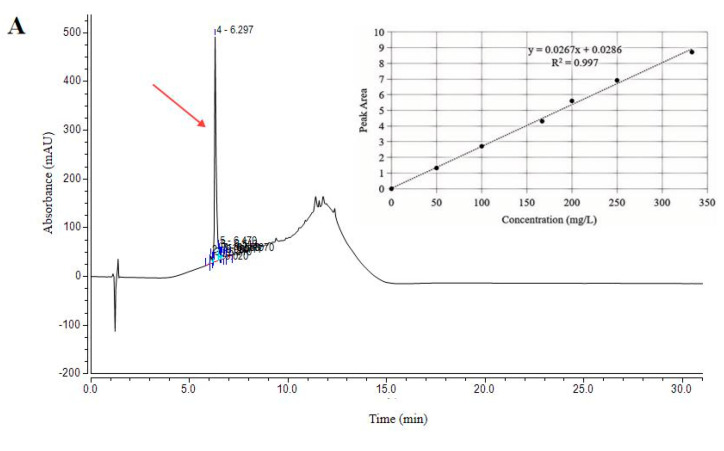

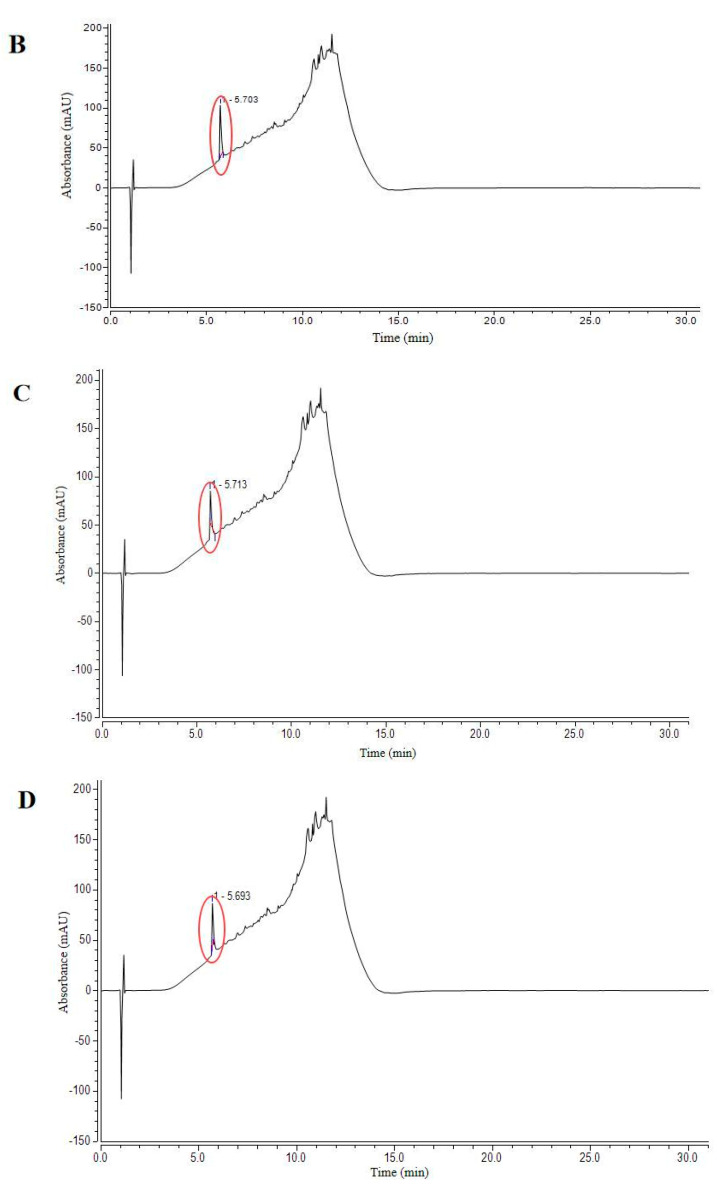
HPLC quantification of thanatin-derived peptides. (**A**) Representative chromatogram of the synthetic thanatin standard (retention time: 6.297 min) and the corresponding standard curve generated from serial dilutions. Overlaid chromatograms of fermentation supernatant samples from the three engineered strain groups: (**B**) PB + Ty (retention time: 5.703 min), (**C**) PB (5.713 min), and (**D**) Ty (5.693 min).

For the quantification of antimicrobial peptide concentration via high-performance liquid chromatography (HPLC), recombinant enterokinase (P4237-1000U) was first applied to cleave the tandem peptide in the fermentation broth, followed by quantification using chemically synthesized antimicrobial peptide as the standard. The results (Figure 9B) showed that the yield of the thanatin-containing chimeric peptide in the fermentation supernatant reached 27.37 ± 3.52 mg/L for the PB + Ty group, 14.56 ± 2.18 mg/L for the PB group (Figure 9C), and 20.36 ± 2.91 mg/L for the Ty group, respectively (Figure 9D). All values are presented as the mean of three independent biological replicates. After enterokinase digestion, the tandem peptide produced two fragments, of which only one carried the EK tag. The EK-tag-free fragment was selected as the analyte for HPLC quantification. However, due to the change in molecular mass after enterokinase cleavage, the concentration determined by this method may deviate from that of the intact tandem peptide. All samples were analyzed in three independent replicates, and the data presented represent the mean values of each experimental group.

### 3.3. In Vitro Antibacterial Activity Results

**Figure 10:**
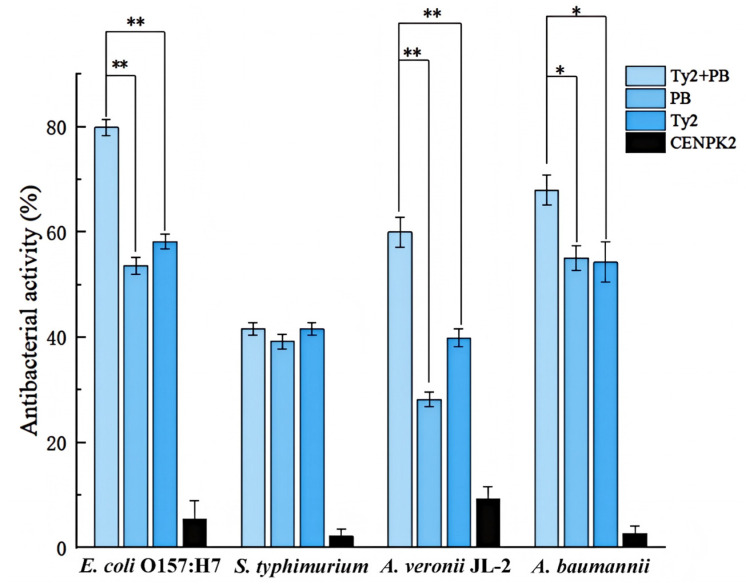
In vitro antibacterial activity of concentrated fermentation supernatants from the recombinant yeast strains against four test bacteria. Note: ** indicates *p* < 0.01. * indicates *p* < 0.05.

The antibacterial activity of concentrated fermentation supernatants against the four indicator strains is shown in Figure 10. Prior to treatment, all bacterial cultures were adjusted to an initial OD_600_ of 0.01, and the 72 h culture broths of the engineered strains were normalized to the same OD_600_ with fresh YPD medium before supernatant collection to ensure comparable amounts of secreted product were tested. The recombinant peptide exhibited inhibitory effects against all four strains. The inhibition rates of the PB and Ty2 groups were higher than that of the negative control, confirming functional heterologous expression of the peptide.

These results demonstrate that the heterologously expressed thanatin-derived peptides are active against all four tested pathogens. From a translational standpoint, this approach could be explored as a strategy to improve the production of antimicrobial peptides in yeast, offering a pathway toward more efficient manufacturing for pharmaceutical or food preservation applications.

The MIC and MBC values of the concentrated fermentation supernatants and the synthetic thanatin standard against the four indicator strains are presented in Table 1. Compared with the thanatin standard, the MIC and MBC values of the four concentrated fermentation supernatants all exhibited a certain degree of reduction, which may be attributed to the fact that the heterologously expressed peptide in the fermentation supernatant is a chimeric peptide consisting of two thanatin moieties and an EK tag, rather than the native monomeric peptide. Notably, MIC and MBC values for some fermentation supernatant samples against indicator strains could not be detected, presumably because the concentration of the target peptide in the fermentation broth was below the MIC/MBC detection threshold for those strains. CENPK2 did not exhibit detectable MIC or MBC values against any of the four indicator strains.

### 3.4. SEM Observations

**Figure 11:**
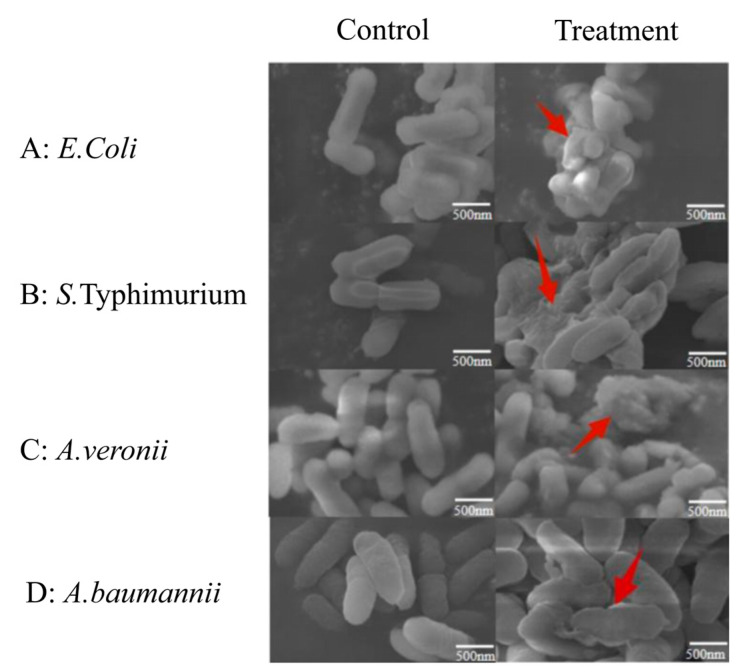
SEM images of four indicator bacteria before and after treatment with thanatin-containing fermentation supernatant from the PB + Ty2 dual-system.

SEM images revealed that untreated bacterial cells displayed smooth, intact surfaces. In contrast, bacteria treated with the concentrated fermentation supernatant from the PB + Ty2 strain showed pronounced morphological alterations, including surface roughening, invagination, pore formation, and cell lysis (Figure 11). The SEM data presented are qualitative in nature and do not provide quantitative measurements.

These observations confirm that the fermentation supernatant induces severe damage to the bacterial cell envelope, consistent with membrane-disrupting effects reported for certain antimicrobial peptides. However, thanatin exerts its antimicrobial activity by targeting both LptA and LptD within this essential transport machinery, thereby disrupting LPS trafficking and outer membrane biogenesis, rather than direct membrane lysis. The observed morphological changes may represent secondary effects following the disruption of outer membrane integrity [21]. Therefore, while the SEM data provide direct visual evidence of the bactericidal action of the recombinant supernatant, they do not by themselves establish the primary mechanism of action of the thanatin-derived peptide. We hypothesize that the chimeric peptide may exhibit different membrane interactions compared with native thanatin, which could potentially explain the observed morphological changes; however, further mechanistic studies are required to confirm this interpretation. From a translational perspective, the morphological assay established here provides a straightforward and accessible approach for future visual screening of antimicrobial efficacy during fermentation optimization. It could also serve as a quality control indicator in bioprocess development, where rapid assessment of bioactivity is beneficial.

### 3.5. Hemolytic Activity Results

**Figure 12:**
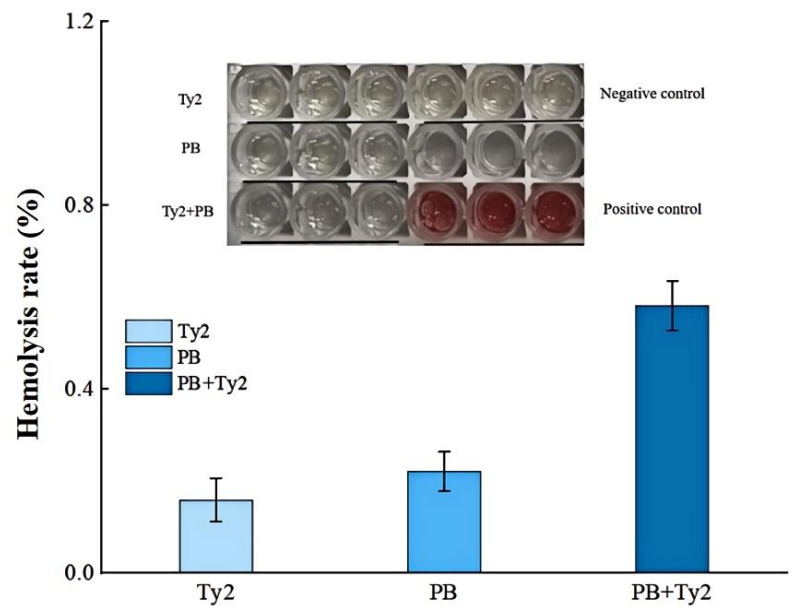
In vitro hemolysis test of physiological saline-replaced fermented supernatants from the recombinant yeast strains on rabbit erythrocytes.

The hemolysis results are shown in Figure 12. The PB, Ty2 and PB + Ty2 fermentation supernatants all exhibited hemolysis rates below 1%, indicating minimal erythrocyte lysis under the tested conditions. This hemolytic activity is substantially lower than that of many conventional antimicrobial peptides; for instance, PGLa exhibits an HC_50_ value of 0.6 μM against human erythrocytes, demonstrating potent hemolytic activity [22].

These data indicate that the fermentation supernatants containing thanatin-derived peptides possess low hemolytic activity, suggesting favorable biocompatibility of the produced peptides. From an application perspective, this low hemolytic profile is an important safety attribute for potential therapeutic or feed-additive uses. However, it should be noted that these results were obtained using crude supernatants rather than purified thanatin; future studies using purified peptide and human erythrocytes will be required to confirm the hemolytic profile more definitively.

## 4. Discussion

In this study, we successfully constructed heterologous expression systems for thanatin in *S. cerevisiae* CENPK2 using the Ty2 and PiggyBac transposon systems, generating engineered strains carrying either system alone or in combination. This transposon-based platform enables stable genomic integration and avoids the instability commonly associated with episomal plasmid-based systems [22].

Thanatin has been primarily produced through chemical synthesis or heterologous expression in *E. coli* and *P. pastoris* [7]. The *S. cerevisiae* system developed in this study offers several distinct advantages: GRAS (Generally Recognized as Safe) status, simple fermentation requirements, absence of endotoxin contamination, and a wealth of well-established genetic tools [14]. The dual-system strain achieved a thanatin yield of up to 27.37 ± 3.52 mg/L in the fermentation supernatant, suggesting that *S. cerevisiae* represents a viable alternative worthy of further investigation. However, *S. cerevisiae* also has limitations, including generally lower protein secretion capacity compared with *P. pastoris* and potential degradation of secreted peptides by yeast-derived proteases during fermentation, which may be addressed through protease-deficient host strains in future studies [23].

Tris-tricine-SDS-PAGE and LC-MS/MS analyses confirmed the successful expression and secretion of thanatin-derived peptides into the culture medium.

Under identical culture conditions, the growth profiles of the Ty2, PB and PB + Ty2 strains were nearly identical to that of the parental strain. This observation suggests that both transposon systems impose minimal metabolic burden on the host [24,25]. The absence of growth impairment also implies that thanatin does not exhibit overt toxicity toward the producer strain, possibly due to the innate tolerance of yeast to cationic antimicrobial peptides, such as cell wall modification and efflux pumps [26].

The concentrated fermentation supernatants from all three engineered strains exhibited significant antibacterial activity against four clinically relevant Gram-negative pathogens: *E. coli* O157:H7, *S. typhimurium*, *A. veronii*, and *A. baumannii* [4]. Notably, the PB + Ty2 dual-system strain consistently demonstrated superior antibacterial activity compared with strains harboring a single transposon system, particularly against *E. coli*, *A. veronii*, and *A. baumannii*. This enhancement may be attributable to increased gene copy number and stable genomic integration achieved by combining two transposition mechanisms; however, this interpretation remains hypothetical until confirmed by quantitative copy-number and expression analyses. Such combinatorial transposon engineering offers a promising strategy for boosting the production yield of difficult-to-express AMPs in yeast. *E. coli* O157:H7 is a major cause of foodborne hemorrhagic colitis and hemolytic uremic syndrome [27]; *S. typhimurium* causes gastroenteritis and systemic infections [28]; *A. veronii* is associated with diarrhea, wound infections, and septicemia in immunocompromised individuals; and *A. baumannii* is a leading nosocomial pathogen that frequently displays multidrug resistance [29]. The ability to inhibit these clinically and agriculturally important pathogens highlights the therapeutic and biopreservative potential of thanatin produced via the transposon-based platform.

Scanning electron microscopy provided direct visual evidence of morphological alterations in treated bacterial cells, including surface roughening, invagination, pore formation, and eventual lysis. These observations are consistent with the membrane-disrupting effects reported for certain AMPs; however, they do not by themselves establish the primary mechanism of action. Given that thanatin is generally considered to act primarily through interference with the LPS transport system (targeting LptA and LptD), the observed membrane damage may represent secondary effects following the disruption of outer membrane integrity. Quantitative assessments, including the percentage of damaged cells and statistical evaluation of morphological alterations, are warranted in future investigations. In addition to its potent antibacterial efficacy, the safety profile of any novel antimicrobial candidate is paramount for therapeutic development. Hemolysis assays revealed that the expressed thanatin exhibited low hemolytic activity (<1%) on rabbit erythrocytes [30], substantially lower than that of many naturally occurring and synthetic AMPs, such as melittin, which has been reported to cause up to 71.63% hemolysis at a concentration of 8 µM [31]. This favorable biocompatibility, observed at the concentration present in the crude supernatant, supports the potential safety of the produced peptide; however, future studies using purified peptide will be necessary for a definitive assessment.

## 5. Limitations and Future Perspectives

While this study demonstrates the successful establishment of a transposon-based expression platform for thanatin, several limitations should be acknowledged. Addressing these limitations will be essential for translating this proof-of-concept platform into a commercially viable production system.

**Quantitative production data.** Although HPLC analysis confirmed and quantified the presence of the chimeric peptide in fermentation supernatants. The thanatin monomer released by enterokinase cleavage and the EK-tagged peptide fragment co-eluted under the chromatographic conditions employed. Since the monomer is approximately half the molecular mass of the tandem construct, owing to co-elution and molecular mass difference, the measured value is a semi-quantitative estimate rather than an absolute quantification of the intact tandem peptide. Its activity and charge properties may be affected [32]. However, as a proof-of-concept, this chimeric peptide is sufficient to demonstrate that the transposon-based expression system can produce active peptides. Furthermore, peptide purification and precise yield determination at various fermentation scales have not yet been performed. Future studies should focus on developing efficient purification protocols.

**Peptide integrity and processing.** LC-MS/MS analysis confirmed the presence of thanatin-derived peptide sequences but did not provide evidence of correct disulfide bond formation or native folding. The expression of thanatin as part of a chimeric tandem peptide with an EK tag may affect its structural conformation and antimicrobial activity. Additionally, the EK tag carries a net negative charge (−3) [33], which may reduce the overall cationic charge of the chimeric peptide and potentially influence its interaction with bacterial membranes. Future studies should employ non-reducing SDS-PAGE, free thiol quantification, or structural analysis (e.g., circular dichroism spectroscopy) to assess disulfide bond formation and native folding. Comparative activity assays between the chimeric and enterokinase-cleaved forms would help determine whether the tag affects bioactivity.

**Proteolytic degradation.** The fermentation supernatant contains a mixture of intact chimeric peptide and degraded fragments. The detection of thanatin-derived peptides following trypsin digestion during LC-MS/MS sample preparation does not necessarily indicate instability during fermentation. However, thanatin’s inherent susceptibility to proteases remains a general concern for small AMPs. To address this, future efforts should explore the use of protease-deficient yeast host strains [34] to minimize degradation during fermentation. Additionally, protein engineering strategies, including site-directed mutagenesis of protease-sensitive residues, backbone cyclization, or incorporation of non-natural amino acids, could be investigated to enhance proteolytic stability while preserving antimicrobial activity.

**Antimicrobial activity assessment.** The antibacterial assays in this study were performed using crude fermentation supernatants rather than purified thanatin. It should be noted that the antimicrobial activity reported herein corresponds to that of the chimeric peptide (two thanatin copies linked by an enterokinase site), not the natural 21-amino acid thanatin. The fermentation supernatant contains a mixture of intact chimeric peptide and degraded fragments; therefore, the observed antimicrobial activity reflects the cumulative effect of this mixture rather than that of the purified peptide. Other yeast-secreted metabolites may also contribute [35]. The use of a control strain partially addresses this issue. However, future studies could employ purified thanatin to further confirm its specific activity, though this is not strictly required for certain applications. Likewise, the MIC and MBC values determined using the crude supernatant may be further refined with purified peptide if necessary.

**Mechanistic evidence for the dual-system advantage.** The enhanced antibacterial activity of the PB + Ty2 dual-system strain suggests a synergistic effect arising from the complementary integration mechanisms of the two systems. Ty2 operates through a “copy-and-paste” mechanism preferentially integrating upstream of Pol III-transcribed genes, while PiggyBac operates through a “cut-and-paste” mechanism integrating into TTAA sites with high precision [36,37]. We hypothesize that the combined use of Ty2 and PB may produce synergy through targeting distinct genomic loci to avoid saturation, complementary integration strategies enabling higher overall gene copy numbers, and reduced competition for integration machinery. The HPLC quantitative data indirectly support this possibility, as the PB + Ty2 strain yielded the highest peptide concentration. However, direct evidence, such as digital PCR-based copy number determination, integration site analysis, and transcriptional profiling, is still required to elucidate this.

**Hemolytic activity.** Hemolysis assays were performed using rabbit erythrocytes and crude supernatant. Although the results indicate low hemolytic activity, the concentration of thanatin in the crude supernatant was not precisely controlled, and only a rough quantification was performed in conjunction with HPLC. Future studies using purified thanatin and human erythrocytes would provide more clinically relevant safety data.

**Scalability and industrial applicability.** The current study represents a proof-of-concept demonstration at the laboratory scale. Key parameters for industrial translation, including fed-batch fermentation performance, large-scale purification efficiency, and cost-effectiveness analysis, have not yet been evaluated. Future work should address these aspects to establish the commercial viability of this platform.

**Peptide purification for activity validation.** A key limitation is that all activity assays were performed using crude fermentation supernatants rather than purified thanatin. Since the supernatant contains other yeast-secreted metabolites that may contribute to or synergize with the observed antimicrobial effects, the activity cannot be unequivocally attributed solely to the recombinant peptide. Future studies should establish a purification protocol (e.g., cation-exchange chromatography) to obtain pure peptide, enabling accurate determination of MIC/MBC values and unambiguous assessment of hemolytic and cytotoxic profiles, thereby providing reliable data for therapeutic evaluation.

## 6. Conclusions

In conclusion, we have established a functional transposon-based proof-of-concept platform for the heterologous expression of a thanatin-containing chimeric peptide in *S. cerevisiae*. The dual transposon system (PB + Ty2) showed enhanced antibacterial activity and higher peptide yield compared with single-system strains under the tested conditions. While the present study provides proof-of-concept validation, further optimization and mechanistic studies will be required to fully evaluate the potential of this platform for practical applications.

## Figures and Tables

**Table 1:** MIC and MBC values of chemically synthesized thanatin standard and concentrated fermentation supernatants from three engineered strain groups.

Strain	Chemically Synthesized Thanatin		The PB + Ty Sample Group		The PB Sample Group		The Ty Sample Group	
	MIC (µg/mL)	MBC (µg/mL)	MIC (µg/mL)	MBC (µg/mL)	MIC (µg/mL)	MBC (µg/mL)	MIC (µg/mL)	MBC (µg/mL)
*E. coli* O157:H7	4	8	2	8	4	8	2	4
*S.* Typhimurium	32	128	32	64	32	>128	32	>128
*A. veronii* JL-2	8	32	8	64	8	64	8	64
*A. baumannii*	256	>1024	>128	>128	>128	>128	>128	>128

## Data Availability

The raw data supporting the conclusions of the article will be made available by the authors upon request.

## Supplementary Materials

The following supporting information can be downloaded at https://www.mdpi.com/article/10.3390/biology15151310/s1, Table S1. Primers used in this study. Figure S1. Original agarose gel electrophoresis images for PCR confirmation of the recombinant strains. Figure S2. Original Tris-tricine-SDS-PAGE analysis of fermentation supernatant.
