# Supplementary material for: Heterologous Expression of Thanatin Using Dual Transposon System in Saccharomyces cerevisiae
Source: Biology (Basel). 2026 Aug 5;15(15):1310. doi: 10.3390/biology15151310 (PMC13464620; doi:10.3390/biology15151310)
Supplement: Supplementary file 1 [file biology-15-01310-s001.zip › biology-4381839-supplementary.pdf]

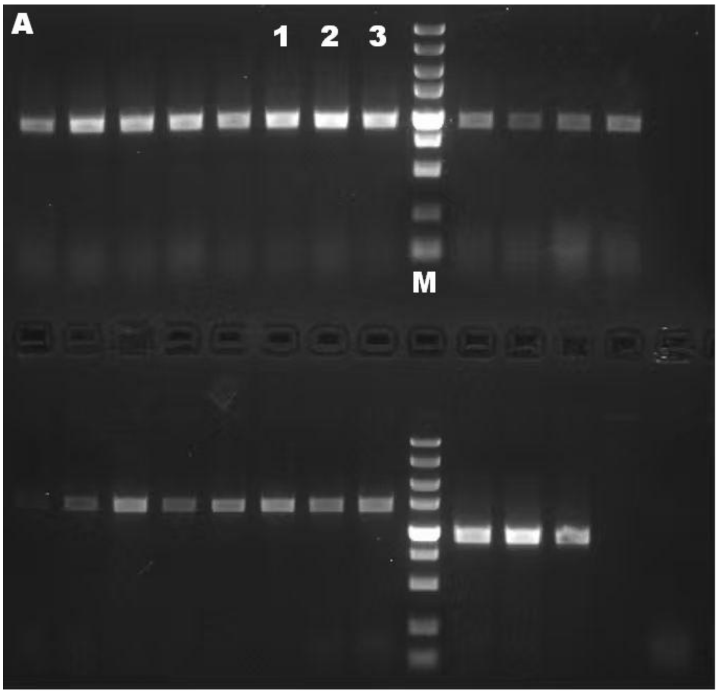

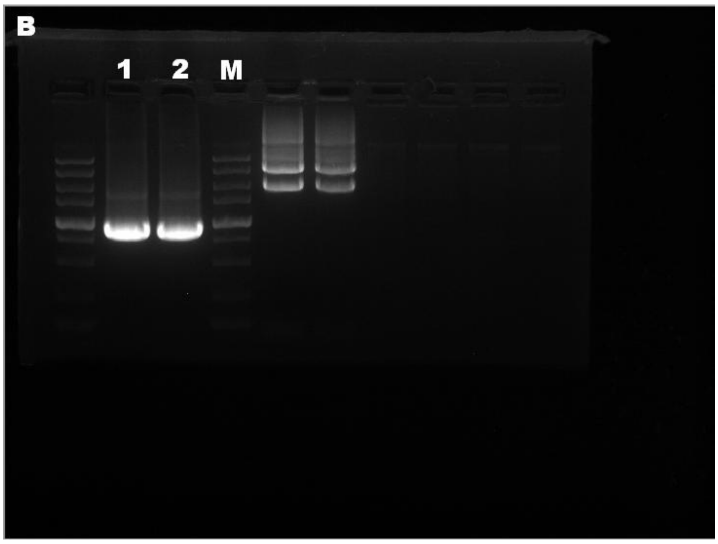

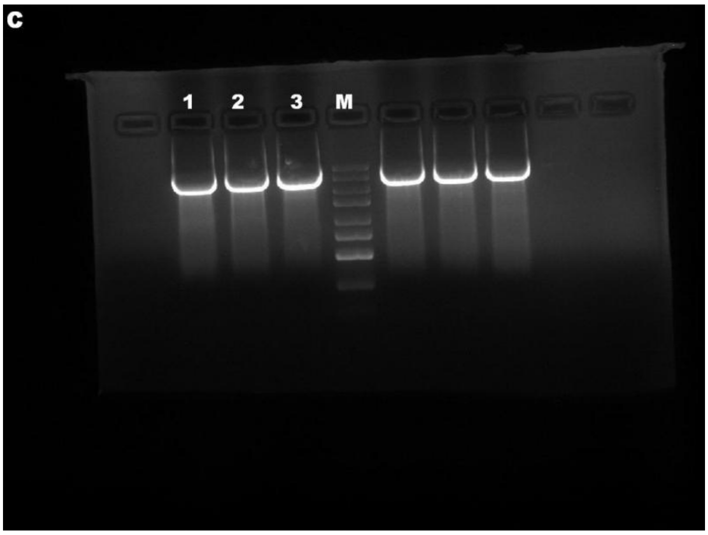

Supplementary Figure S1. Original agarose gel electrophoresis images for PCR confirmation of the

recombinant strains. (**A**) pUC57-Ty2-thanatin; (**B**) PiggyBac transposons; (**C**) pBluescript-piggyITR-thanatin. M: DNA marker (top to bottom: 5000, 3000, 2000, 1500, 750, 500, 250, 100 bp); lanes 1–3: independent transformants. The original uncropped gels are presented to ensure full transparency of the data.

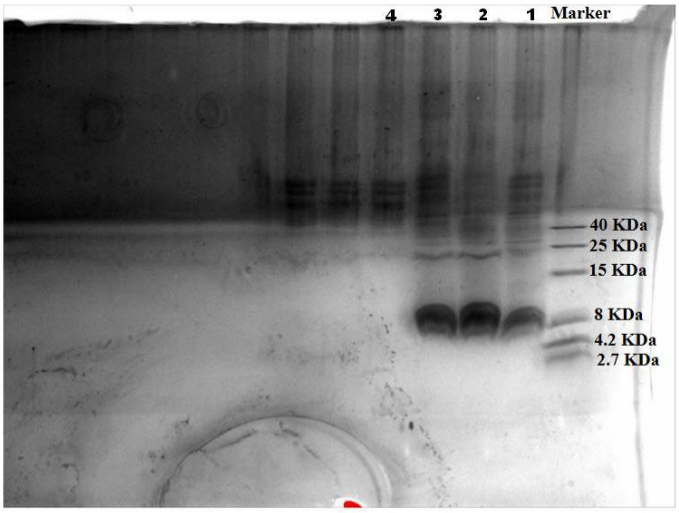

Figure S2. Tris-tricine-SDS-PAGE analysis of fermentation supernatants. From right to left: Marker: protein marker; Lane 1, PB+Ty; Lane 2, PB; Lane 3, Ty; Lane 4, CENPK2 (control)

Table S1. Primers used in this study.

Primer Name	Sequence (5' -> 3')	Purpose
Ty2-ITR-F	AAGAACATTGCTGATGTGATG	To obtain Ty2 retrotransposon long terminal repeat (LTR) sequence.
Ty2-ITR-R	CATCAAAATCCACATTCTCT	
Ty2-upstream-F(Gib）	TTGTAAAACGACGGCCAGTGGCGGCCGCTGTTGGAATAAAAATCAACTATCATCTAC	To obtain the upstream and upstream homologous arms of the Ty2 LTR for Gibson Assembly.
Ty2-upstream-R(Gib）	GAGGTCGCTCGTTAATATTCATTGATCCTATTACATTATCAATC	
Ty2-downstream-F(Gib）	GATCCACTAGTGCCGCGGATATAAAATGATGATAATAATATTTATAGAATTGTG	To obtain the upstream and downstream homologous arms of the Ty2 LTR for Gibson Assembly.
Ty2-downstream-F(Gib）	CTATGACCATGATTACGCCAGCGGCCGCGAGAATGTGGATTTTGATGTAATTG	
pTEF1-F(Gib)	TCTGCAGTCGACGGGCCCGGAGTGATCCCCCACACACCATAG	To obtain expression cassette for Gibson Assembly
tADH1-R(Gib)	GAATATTAACGAGCGACCTCATGCTATAC	
Tpos-F(Gib)	AATAAACACACATAAACAAACAAAGATGGGTTCCTCCTTGGATGATGAACATATTTTG	To obtain the coding sequence of the hyperactive PiggyBac transposase for use in Gibson assembly
Tpos-R(Gib)	CGAAGAATTGTTAATTAAGAGCTCTAGTGGTGATGGTGATGATGGAAGCAGGATTGA	
PB-3′ ITR-F(Gib)	GTTTTCCCAGTCACGACGTTG	To obtain the homologous arm of the PiggyBac 3′-ITR for use in Gibson assembly。
PB-3′-ITR-R(Gib)	TTGCATGCAGGCCTCTGCAGTTTTCCCCGTATCCCCCCAGGTGTCTGCAGG	

PB-5′ITR-F(Gib)	GCGAATGCATCTAGATATCGTTAACCCTAGAAAGATAGTCTGCGTAAAATTGACGC	To obtain the homologous arm of the PiggyBac 5′-ITR for use in Gibson assembly。
**PB-5′-ITR-R**(Gib)	GGGGGCTGTCCCTCTAGCCTGATATCTATAACAAGAAAATATATATATAA	
